# FEAR AND DYNAMIC INFLAMMATORY RESPONSE TO MOVEMENT AMONG OLDER ADULTS WITH LOW BACK PAIN: A FEASIBILITY ANALYSIS

**DOI:** 10.1093/geroni/igz038.2629

**Published:** 2019-11-08

**Authors:** Corey Simon, Francis Keefe, Gregory Hicks, Carl Pieper, Cathleen Colon-Emeric

**Affiliations:** 1 Duke University, Durham, North Carolina, United States; 2 University of Delaware, Newark, Delaware, United States; 3 Duke University Medical Center, Durham, North Carolina, United States

## Abstract

Low back pain (LBP) is a prevalent cause of disability, with higher prevalence among older adults. However, the level of LBP disability cannot be explained by either pain intensity or LBP pathology (bone degeneration). We hypothesize that older adults with LBP are susceptible to disability based on the combination of two factors: fear of movement and dynamic inflammatory response to movement (DIR). In this feasibility study, we are measuring these two factors among 30 older adults aged 60-85 with LBP. Participants attend a laboratory session to complete four performance tasks: 30-second chair rises, seated trunk rotation, standing forward reach, and Six-Minute Walk. Fear of movement is tested using the Tampa Scale for Kinesiophobia and Situational Catastrophizing Questionnaire. DIR is tested by drawing blood via a peripheral venous catheter before and after performance tasks, and assessing changes in six inflammatory markers: IL-6,8,10; TNF-alpha, c-reactive protein, and substance P. LBP disability is assessed using the Late-life Functional Disability Instrument (LLFDI) and 7-day ecological momentary assessment (EMA) via smartphone application. Participants have tolerated all laboratory testing procedures and have adequately reported LBP disability in the home and community using EMA. We will test for exploratory associations between fear of movement, DIR, and LBP disability measures using ordinary least squares regression analyses. The overarching goal of this research line is to determine if the combination of fear of movement and DIR are an actionable clinical risk phenotype of downstream LBP disability among older adults.

